# MAGE expression in head and neck squamous cell carcinoma primary tumors, lymph node metastases and respective recurrences-implications for immunotherapy

**DOI:** 10.18632/oncotarget.14830

**Published:** 2017-01-27

**Authors:** Simon Laban, Gregor Giebel, Niklas Klümper, Andreas Schröck, Johannes Doescher, Giulio Spagnoli, Julia Thierauf, Marie-Nicole Theodoraki, Romain Remark, Sacha Gnjatic, Rosemarie Krupar, Andrew G. Sikora, Geert Litjens, Niels Grabe, Glen Kristiansen, Friedrich Bootz, Patrick J. Schuler, Cornelia Brunner, Johannes Brägelmann, Thomas K. Hoffmann, Sven Perner

**Affiliations:** ^1^ Department of Oto-Rhino-Laryngology and Head and Neck Surgery, Head and Neck Cancer Center Ulm, University Medical Center Ulm, Ulm, Germany; ^2^ Pathology of the University Medical Center Schleswig-Holstein, Campus Luebeck and the Research Center Borstel, Leibniz Center for Medicine and Biosciences, Germany; ^3^ Department of Otorhinolaryngology, University Hospital Bonn, Bonn, Germany; ^4^ Department of Biomedicine, University Hospital Basel, Basel, Switzerland; ^5^ Department of Medicine, Hematology and Medical Oncology, Icahn School of Medicine at Mount Sinai, Mount Sinai Hospital, New York City, NY, USA; ^6^ Department of Otolaryngology-Head and Neck Surgery, Baylor College of Medicine, Houston, TX, USA; ^7^ Hamamatsu Tissue Imaging and Analysis Center, BIOQUANT, University of Heidelberg, Heidelberg, Germany; ^8^ Institute of Pathology, University Hospital Bonn, Bonn, Germany; ^9^ Department of Oncology, University Hospital Bonn, Bonn, Germany

**Keywords:** cancer-testis antigens, MAGE, melanoma-associated antigen, head and neck squamous cell carcinoma, HNSCC

## Abstract

Melanoma associated antigens (MAGE) are potential targets for immunotherapy and have been associated with poor overall survival (OS) in head and neck squamous cell carcinoma (HNSCC). However, little is known about MAGE in lymph node metastases (LNM) and recurrent disease (RD) of HNSCC.

To assess whether MAGE expression increases with metastasis or recurrence, a tissue microarray (TMA) of 552 primary tumors (PT), 219 LNM and 75 RD was evaluated by immunohistochemistry for MAGE antigens using three monoclonal antibodies to multiple MAGE family members. Mean expression intensity (MEI) was obtained from triplicates of each tumor specimen.

The median MEI compared between PT, LNM and RD was significantly higher in LNM and RD. In paired samples, MEI was comparable in PT to respective LNM, but significantly different from RD. Up to 25% of patients were negative for pan-MAGE or MAGE-A3/A4 in PT, but positive in RD. The prognostic impact of MAGE expression was validated in the TMA cohort and also in TCGA data (mRNA). OS was significantly lower for patients expressing pan-MAGE or MAGE-A3/A4 in both independent cohorts.

MAGE expression was confirmed as a prognostic marker in HNSCC and may be important for immunotherapeutic strategies as a shared antigen.

## INTRODUCTION

Head and neck squamous cell carcinoma (HNSCC) of the oral cavity, pharynx and larynx combined account for 60,000 new cases of malignant tumors and 12,000 tumor related deaths annually in the USA, with comparable numbers in Europe [1–3]. The prognosis of HNSCC patients remains unsatisfactory with 5-year overall survival rates of approximately 50–65% without significant improvement in the last decades [4]. Recent advances in the field of cancer immunology and cancer immunotherapy imply that immunotherapy may be key to improve this outcome. Notably, a subset of HNSCC patients treated with checkpoint blockade therapy experience clinical benefit [5–7]. Characterizing tumor antigens recognized by the immune system and potentially targeted during immunotherapy is therefore likely to yield important biomarkers of prognostic or predictive value. Cancer-testis antigens (CTA) are a group of immunogenic tumor antigens, which can be specifically detected in different cancers, but not in healthy normal tissue except for human testis [8, 9]. Their restricted expression and immunogenicity make them good candidates to assess as shared targets of immunotherapy.

Melanoma-associated antigens (MAGE) are among the most frequently expressed CTA in HNSCC [10–13]. We previously demonstrated in a large cohort of surgically treated HNSCC patients that expression of pan-MAGE and MAGE-A3/A4 is associated with reduced overall survival (OS). The prognostic value was strongest if the staining pattern was considered. Simultaneous cytoplasmic (cyt) and nuclear (nuc) antigen expression (cyt+nuc) was identified as an independent prognostic marker [10].

Because MAGE expression is often heterogeneous within primary tumors (PT), dynamic changes and clonal evolution of MAGE expression should be also assessed upon tumor progression. Yet, very little is known about MAGE expression in lymph node metastases (LNM) or recurrent disease (RD) compared to PT. Since the most frequent types of treatment failure are local and regional recurrences [14], it is important to correlate expression of MAGE in primary tumors, lymph node metastases, and recurrent tumors within the same patients.

Mechanistic data from bladder cancer imply that MAGE-A3 is predominantly expressed in a cancer stem cell like subpopulation [15]. Further mechanistic data from HNSCC cell lines showed reduced treatment efficacy in MAGE expressing cell lines treated with different chemotherapeutics [16]. These factors would suggest an enrichment of MAGE expressing cancer cells during cancer evolution, especially after unsuccessful cancer treatment. At the same time, immune responses to MAGE have been described in HNSCC and may result in reduced MAGE expression during tumor evolution [17]. Based on these previous mechanistic studies, we hypothesized that MAGE expression may underlie a selection pressure during tumor evolution resulting in differential expression in primary tumors, respective lymph node metastases and recurrences. We took advantage of a unique large set of patients with paired tissues collected from primaries, metastases, and recurrences, and used them to assess MAGE evolution and to validate the prognostic impact of MAGE expression in independent HNSCC cohorts.

## RESULTS

### Patient cohort

The cohort comprised 129 oral cavity, 175 oropharyngeal, 66 hypopharyngeal and 182 laryngeal primaries. Mean OS was 105.4 months, mean recurrence-free survival (RFS) was 82.2 months. The majority of patients were treated surgically (*n* = 458), 74 patients were treated by primary chemoradiation and 20 patients were treated with palliative intention. Clinico-pathological criteria of the patient cohort separately for each primary site can be found in Table 1.

**Table 1:** Patient characteristics of the tissue microarray cohort

		Primary Site									
		Oral Cavity (23%)		Oropharynx (31%)		Hypopharynx (12%)		Larynx (33%)		Total Cohort (100%)	
Count	%	Count	%	Count	%	Count	%	Count	%
T	1	50	38,8%	32	18,3%	7	10,6%	80	44,0%	169	30,6%
	2	52	40,3%	59	33,7%	20	30,3%	41	22,5%	172	31,2%
	3	13	10,1%	54	30,9%	13	19,7%	38	20,9%	118	21,4%
	4	11	8,5%	30	17,1%	25	37,9%	23	12,6%	89	16,1%
	missing	3	2,3%	0	0,0%	1	1,5%	0	0,0%	4	0,7%
	total	129	100,0%	175	100,0%	66	100,0%	182	100,0%	552	100,0%
N	0	74	57,4%	41	23,4%	14	21,2%	140	76,9%	269	48,7%
	1	22	17,1%	34	19,4%	14	21,2%	8	4,4%	78	14,1%
	2a	5	3,9%	10	5,7%	6	9,1%	6	3,3%	27	4,9%
	2b	20	15,5%	50	28,6%	19	28,8%	15	8,2%	104	18,8%
	2c	4	3,1%	29	16,6%	8	12,1%	10	5,5%	51	9,2%
	3	0	0,0%	8	4,6%	2	3,0%	1	0,5%	11	2,0%
	missing	4	3,1%	3	1,7%	3	4,5%	2	1,1%	12	2,2%
	total	129	100%	175	100%	66	100%	182	100%	552	100%
M	0	125	96,9%	167	95,4%	64	97,0%	173	95,1%	529	95,8%
	1	3	2,3%	7	4,0%	2	3,0%	9	4,9%	21	3,8%
	missing	1	0,8%	1	0,6%	0	0,0%	0	0,0%	2	0,4%
	total	129	100%	175	100%	66	100%	182	100%	552	100%
Grading	1	11	9%	3	2%	0	0%	6	3%	20	4%
	2	72	56%	99	57%	25	38%	95	52%	291	53%
	3	26	20%	45	26%	28	42%	33	18%	132	24%
	missing	20	16%	28	16%	13	20%	48	26%	109	20%
	total	129	100%	175	100%	66	100%	182	100%	552	100%
HPV status (DNA)	HPV DNA negative	123	96,1%	149	87,1%	63	95,5%	178	97,8%	513	93,0%
	HPV DNA positive	6	4,7%	26	15,2%	3	4,5%	4	2,2%	39	7,0%
	total	129	100,8%	175	102,3%	66	100,0%	182	100,0%	552	100,9%
HPV status (DNA + p16)	HPV negative	123	96,1%	151	88,3%	64	97,0%	182	100,0%	520	95,1%
	HPV positive	5	3,9%	20	11,7%	2	3,0%	0	0,0%	27	4,9%
	missing	1	0,8%	4	2,3%	0	0,0%	0	0,0%	5	0,9%
	total	128	100,0%	171	100,0%	66	100,0%	182	100,0%	547	100,0%
treatment approach	surgical	116	89,9%	137	78,3%	54	81,8%	151	83,0%	458	83,0%
	non-surgical	1	0,8%	32	18,3%	11	16,7%	30	16,5%	74	13,4%
	other (non-curative)	12	9,3%	6	3,4%	1	1,5%	1	0,5%	20	3,6%
	total	129	100,0%	175	100,0%	66	100,0%	182	100,0%	552	100,0%
sex	male	75	58,1%	132	23,3%	56	9,7%	154	27,6%	417	75,5%
	female	54	41,9%	43	7,8%	10	1,8%	28	5,1%	135	24,5%
	total	129	100,0%	175	100,0%	66	100,0%	182	100,0%	552	100,0%
smoking_><median (36)	never smoker	12	9,3%	22	12,6%	4	6,1%	11	6,0%	49	8,9%
	<36 py	24	18,6%	53	30,3%	25	37,9%	65	35,7%	167	30,3%
	>=36py	36	27,9%	73	41,7%	27	40,9%	81	44,5%	217	39,3%
	missing	57	44,2%	27	15,4%	10	15,2%	25	13,7%	119	21,6%
	total	129	100,0%	175	100,0%	66	100,0%	182	100,0%	552	100,0%

### MAGE protein expression frequency in primary tumors, lymph node metastases and recurrences

**Figure 1:**
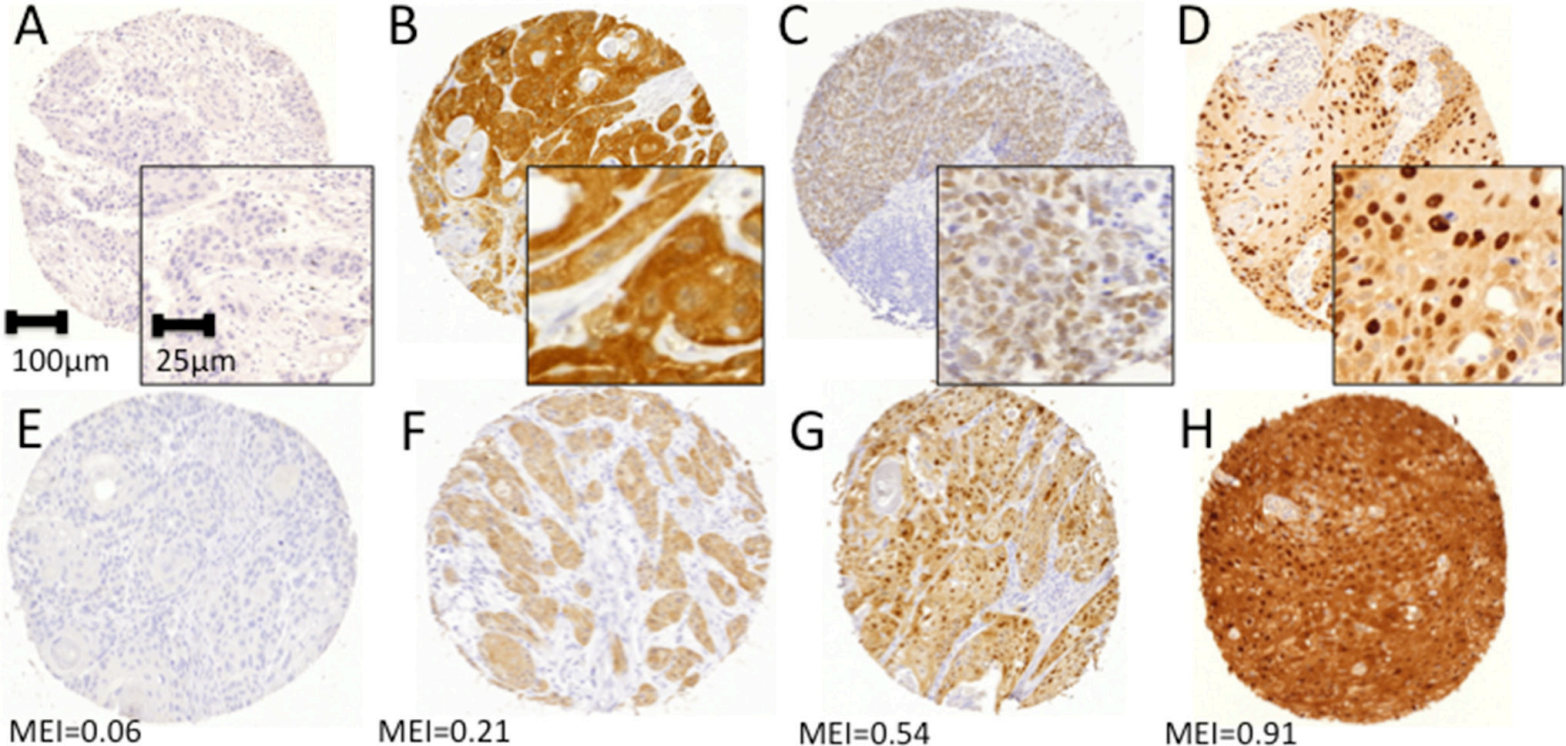
Representative immunohistochemical staining examples for pan-MAGE (M3H67) Different staining patterns were attributed visually by two independent observers. (**A**) Negative sample, (**B**) cytoplasmic expression, (**C**) nuclear expression, (**D**) combined cytoplasmic and nuclear expression. Digital pathology was used to determine the expression intensity of each core. Examples for different expression intensities are presented with the respective mean expression intensity indicated below (**E**–**H**).

As previously reported, we observed three different expression patterns of MAGE for pan-MAGE (M3H67) and MAGE-A3/A4 (57B) expression: cyt, nuc and cyt+nuc. MAGE-A1 (MA454) is always solely expressed cytoplasmically [10]. Representative staining examples of negative samples and the different expression patterns (cyt, nuc, cyt+nuc) are given in Figure 1A–1D. Additionally, examples for different mean expression intensities (MEI) are presented in Figure 1E–1H.

Visual scoring of antigen expression and expression pattern was performed microscopically by two independent analysts. The expression was considered positive if any of the three triplicates for each sample stained positive for the respective antigen. Pan-MAGE was found in 173/ 423 (41%) of primary tumors, 97/212 (46%) of lymph node metastases and 18/49 (37%) of recurrences respectively. MAGE-A3/A4 was positive in 166/423 (39%) of primary tumors, 90/212 (42%) of lymph node metastases and 18/49 (37%) of recurrences. MAGE-A1 was less frequently found with 78/424 (18%) in primary tumors, 38/212 (18%) in lymph node metastases and 12/49 (25%) in recurrent tumors. Notably, the frequency of combined cyt+nuc expression seems to be higher in RD. Detailed numbers and the distribution of the expression pattern for each antigen are presented in Table 2.

**Table 2:** MAGE expression patterns by sample type, primary tumor site and HPV status

		pan-MAGE (M3H67)			Chi2 (2-sided)	MAGE-A3/A4 (57B)			Chi2 (2-sided)	MAGE-A1 (MA454)		Chi2 (2-sided)
		negative	cyt OR nuc	cyt + nuc		negative	cyt OR nuc	cyt + nuc		negative	positive	
Primary Tumor		250 (59.1%)	91 (21.2%)	82 (19.4%)	n.a.	258 (60.9%)	78 (18.4%)	87 (20.6%)	n.a.	346 (82%)	78 (18%)	n.a.
Lymph Node Metastasis		115 (54.2%)	51 (24.1%)	46 (21.7%)		122 (57.5%)	34 (16.0%)	56 (26.4%)		174 (82%)	38 (18%)	
Recurrent Disease		31 (63.3%)	5 (10.2%)	13 (26.5%)		31 (63.3%)	8 (16.3%)	10 (26.4%)		37 (76%)	12 (25%)	
Oral Cavity		67 (68.4%)	19 (19.3%)	12 (12.2%)	p=0.043*	69 (70.4%)	19 (19.4%)	10 (10.2%)	p=0.039*	90 (91.8%)	8 (8.2%)	p=0.002**
Oropharynx		82 (58.6%)	24 (17.1%)	34 (24.3%)		84 (60.4%)	26 (18.7%)	29 (20.8%)		112 (80.0%)	28 (20.0%)	
Hypopharynx		24 (44.5%)	16 (29.6%)	14 (25.9%)		28 (51.8%)	14 (25.9%)	12 (22.2%)		36 (66.7%)	18 (33.3%)	
Larynx		77 (58.8%)	32 (24.2%)	22 (16.7%)		77 (58.3%)	19 (14.3%)	36 (27.3%)		108 (81.8%)	24 (18.2%)	
Non-Oropharynx		168 (59.4%)	67 (23.6%)	48 (16.9%)	p=0.108	174 (61.2%)	52 (18.3%)	58 (20.4%)	p=0.986	234 (82.3%)	50 (17.6%)	p=0.550
Oropharynx		82 (58.6%)	24 (17.1%)	34 (24.3%)		83 (59.7%)	26 (18.7%)	29 (20.8%)		112 (80.0%)	28 (20.0%)	
HPV (DNA)	negative	222 (57.4%)	84 (21.7%)	81 (20.9%)	p=0.018*	228 (58.9%)	74 (19.1%)	85 (21.9%)	p=0.013*	313 (80.6%)	75 (19.4%)	p=0.119
	positive	28 (77.8%)	7 (19.4%)	1 (2.7%)		30 (83.3%)	4 (11.1%)	2 (5.5%)		33 (91.7%)	3 (8.3%)	
HPV (DNA/p16)	negative	225 (57.3%)	86 (21.8%)	82 (20.8%)	p=0.002**	232 (59.0%)	76 (19.3%)	85 (21.6%)	p=0.011*	318 (80.7%)	76 (19.3)	p=0.063
	positive	24 (92.3%)	2 (7.7%)	0 (0%)		23 (88.5%)	1 (3.8%)	2 (7.6%)		25 (96.2%)	1 (3.9%)	

*significant at 0.05 level; ** significant at 0.01 level; *** significant at 0.001 level. n.a. not applicable

### Impact of primary site and HPV status on MAGE expression

MAGE expression frequency in primary tumors was significantly different based on the primary site for pan-MAGE (*p* = 0.04), MAGE-A3/A4 (*p* = 0.04) and MAGE-A1 (*p* = 0.002) whereas the lowest expression rate was found in oral cavity primaries and the highest expression rate in hypopharyngeal primaries. MAGE expression was less frequently found in HPV positive cases based on HPV-DNA for pan-MAGE (HPV-DNA_negative_/pan-MAGE_positive_: 165/387 (42.6%), HPV-DNA_positive_/pan-MAGE_positive_: 8/36 (22.2%) *p* = 0.018) as well as for MAGE-A3/A4 (HPV-DNA_negative_/MAGE-A3/A4_positive_: 159/387 (41.0%), HPV-DNA_positive_/MAGE-A3/A4_positive_: 6/36 (16.7%), *p* = 0.013). Based on HPV DNA and p16 status similar results were found for pan-MAGE (HPV(DNA+p16)_negative_/pan-MAGE_positive_ 168/393 (42.7%), HPV(DNA+p16)_positive_/pan-MAGE_positive_ 2/26 (7.7%), *p =* 0.002) and MAGE-A3/A4 (HPV(DNA+p16)_negative_/ MAGE-A3/A4_positive_ 161/393 (40.9%), HPV(DNA+p16)_positive_/pan-MAGE_positive_ 3/26 (11.5%), *p* = 0.011). Detailed numbers including the distribution of the expression pattern for MAGE positive cases are provided in Table 2.

### MAGE expression intensity differs between primaries, lymph node metastases, and recurrences

We first analyzed data in each type of samples (PT, LNM, RD), focusing only on cases with MEI ≥ 0.1, and found that the median MEI of the groups differed. Pan-MAGE: PT median MEI = 0.24 (*n* = 120), LNM median MEI = 0.36 (*n* = 68), RD median MEI = 0.37 (*n* = 14). MAGE-A3/A4: primary PT median MEI = 0.27 (*n* = 101), LNM median MEI = 0.31 (*n* = 67), RD median MEI = 0.40 (*n* = 12). MAGE-A1: PT median MEI=0.21 (*n* = 26), LNM median MEI = 0.26 (*n* = 20), RD median MEI = 0.33 (*n* = 7).

**Figure 2:**
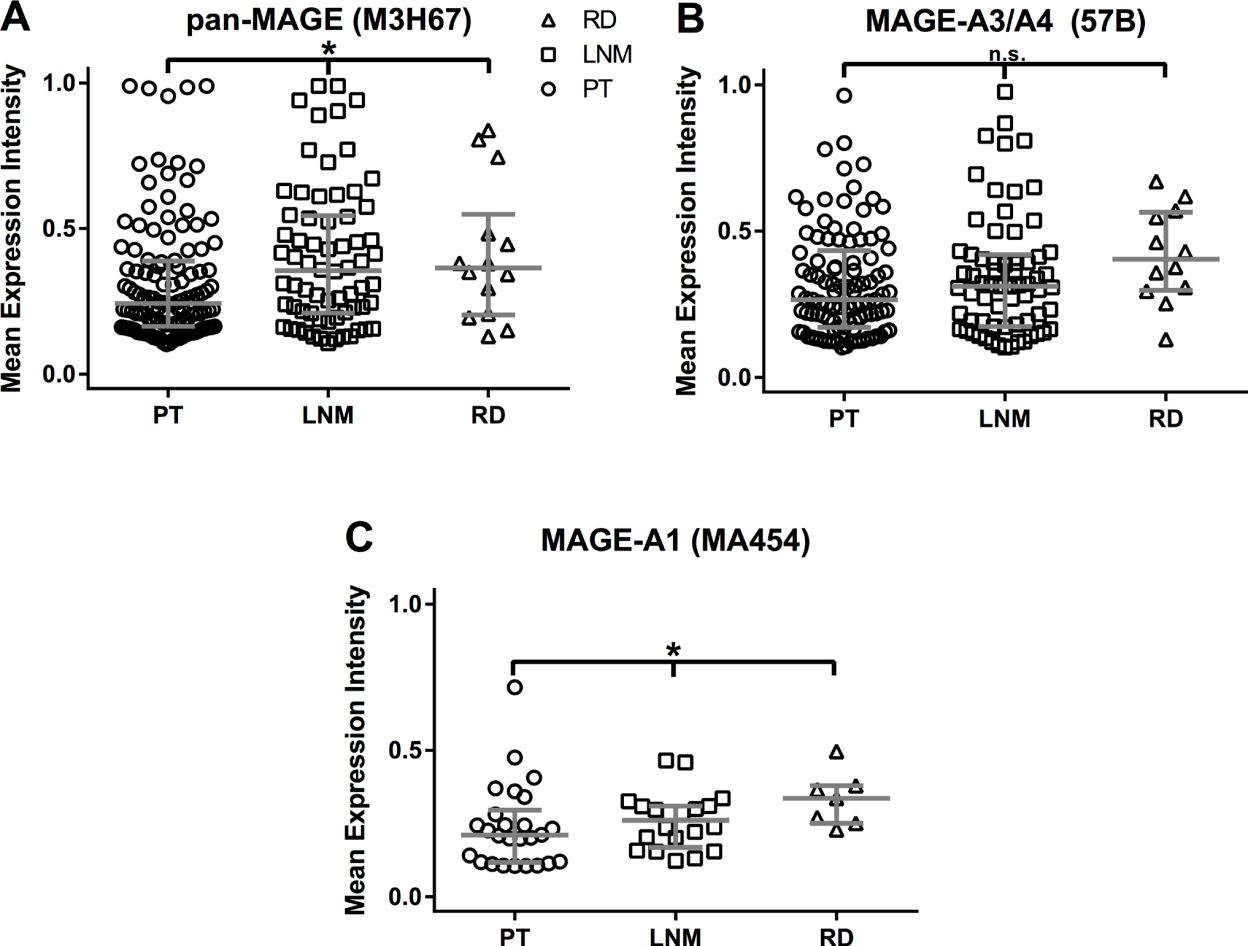
Mean Expression Intensities (MEI) in samples of primary tumors (P), lymph node metastases (LNM) and recurrenct disease (RD) All available samples with a MEI ≥ 0.1 were included in this analysis. MEI values are shown for each triplicate. The horizontal bar indicates the median MEI, the vertical bars indicate the interquartile range. Median MEI were as follows: Pan-MAGE: *P* = 0.24 (*n* = 120), L = 0.36 (*n* = 68), *R* = 0.37 (*n* = 14). MAGE-A3/A4: *P* = 0.27 (*n* = 101), L = 0.31 (*n* = 67), *R* = 0.40 (*n* = 12). MAGE-A1: *P* = 0.21 (*n* = 26), L = 0.26 (*n* = 20), *R* = 0.33 (*n* = 7). By Kruskal Wallis test the median MEI were different for pan-MAGE (*p* = 0.018) and MAGE-A1 (*p* = 0.041), but did not reach significance for MAGE-A3/A4 (*p* = 0.111).

The MEI medians of the respective groups (primary tumors, lymph node metastases, recurrences) were compared by Kruskal-Wallis test. For pan-MAGE (Kruskal-Wallis: 8.06, *p* = 0.018) and MAGE-A1 (Kruskal-Wallis: 6.40, *p* = 0.041), a significant difference between the median MEI was found. The differences between the median MEI for MAGE-A3/A4 expression did not reach significance (Kruskal-Wallis: 4.39, *p* = 0.111). MEI distribution and the median MEI with interquartile range for the three groups (PT, LNM, RD) are depicted for each antigen in Figure 2.

### MAGE expression intensity distribution is concordant between paired samples of primaries and lymph node metastases, but discordant comparing primaries and respective recurrences

In order to compare the MEI between primary tumors (PT), lymph node metastases (LNM) and recurrent disease (RD) in individual patients, paired samples were analyzed. Paired samples were compared using Wilcoxon signed rank test for related samples.

**Figure 3:**
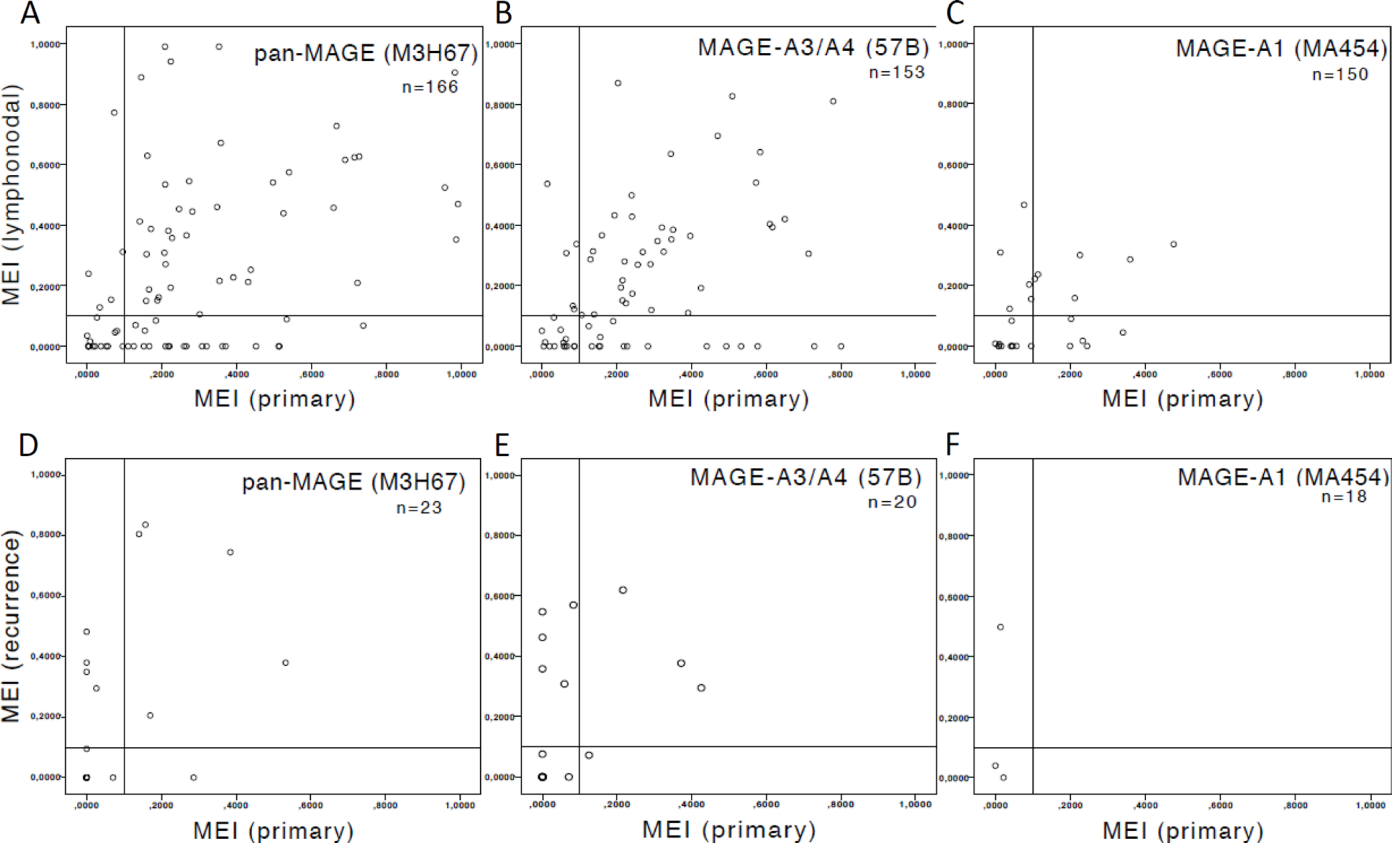
Scatter plot of Mean Expression Intensities (MEI) in paired samples The available number of samples (*n*) is indicated. The threshold of MEI = 0.1 for primary tumors (PT) is indicated by a vertical bar and a horizontal bar for lymph node metastases (LNM) and recurrent disease (RD) respectively. MEI was compared in related samples using Wilcoxon signed rank test. (**A**–**C**) depict MEI of paired samples of PT and LNM. The median of differences between the MEI of PT and respective LNM was not different for (A) pan-MAGE (*p* = 0.900), (B) MAGE-A3/A4 (*p* = 0.594) or (C) MAGE-A1 (*p* = 0.601). (**D**–**F**) depict MEI of paired samples of PT and RD. Significant differences were observed for (D) pan-MAGE (*n* = 23, *p* = 0.034), (E) MAGE-A3/A4 (*n* = 20, *p* = 0.041), but not for (F) MAGE-A1 (*n* = 18, *p* = 0.144). The number of discordant samples in the upper left quadrant (PT = negative, RD = positive; pan-MAGE: 17.4%, MAGE-A3/A4: 25%, MAGE-A1: 16.7%) is higher than in the lower right quadrant (PT = positive, RD = negative; pan-MAGE: 4.3%, MAGE-A3/A4: 5%, MAGE-A1: 0%)).

The median of differences between the MEI of PT and respective LNM was not different for pan-MAGE (*n* = 166, *p* = 0.900), MAGE-A3/A4 (*n* = 153, *p* = 0.594) or MAGE-A1 (*n* = 150, 0.601). Individual MEI values for each paired sample are depicted in Figure 3A–3C. The graph is divided into four quadrants by a cut-off of MEI = 0.1. The lower left quadrants (Q1: PT and LNM negative (MEI < 0.1)) and upper right quadrants (Q3: PT positive and LNM positive (MEI ≥ 0.1)) show concordant samples. The upper left quadrants (Q2: PT negative (MEI < 0.1) and LNM positive (MEI ≥ 0.1)) and lower right quadrants (Q4: PT positive (MEI ≥ 0.1) and LNM negative (MEI < 0.1)) show discordant samples. Most related sample MEI values are concordant: pan-MAGE: 129/166 (77.7%); MAGE-A3/A4: 119/153 (77.8%); MAGE-A1: 136/150 (90.7%)). For pan-MAGE, 37/166 (22.3%) cases showed discordant expression between PT and LNM (Q2: 15/166 (9%); Q4: 22/166 (13.3%)). For MAGE-A3/A4, 34/153 (22.2%) (Q2: 19/153 (12.4%); Q4: 15/153 (9.8%)) and for MAGE-A1 14/150 (9.3%) (Q2: 9/150 (6%); Q4: 5/150 (3.3%) cases respectively were discordant between PT and LNM.

Comparing the median of differences between the MEI of PT and respective RD significant differences were observed for pan-MAGE (*n* = 23, *p* = 0.034), MAGE-A3/A4 (*n* = 20, *p* = 0.041), but not for MAGE-A1 (*n* = 18, *p* = 0.144). Individual MEI values for each paired sample are depicted in Figure 3D–3F. The graph is also divided into four quadrants (by a cut-off of MEI = 0.1 for PT and RD respectively. This graph shows a higher number of RD with MEI ≥ 0.1 that showed MEI < 0.1 in the respective PT (upper left quadrant: pan-MAGE: 4/23 (17.4%); MAGE-A3/A4: 5/20 (25%); MAGE-A1: 3/18 (16.7%)) than the other way around (lower right quadrant: pan-MAGE: 1/23 (4.3%); MAGE-A3/A4: 1/20 (5%); MAGE-A1: 0/18 (0%)).

### Prognostic parameters

Known prognostic markers were evaluated for their impact on OS by Cox regression analysis. The analysis was performed for the whole cohort with the following known prognostic parameters: T-, N- and M-classification, grading, primary treatment, human papilloma virus (HPV) status, sex, primary site and smoking habits. Results are presented in Table 3. Cox regression analysis revealed the highest HR for the primary treatment (surgical vs. non-surgical; HR = 3.012 (95% CI 2.189–4.146, *p* < 0.001). This can be attributed to the treatment algorithms in Germany. Most centers and the majority of patients prefer primary surgical treatment in the curative setting for eligible patients. Only a minority of resectable patients receive primary (chemo-)radiotherapy. Thus, elderly patients, patients with high comorbidities or unresectable cancers are overrepresented in the non-surgical cohort. To eliminate this confounder, the following known prognostic parameters were separately evaluated in the cohort of patients who received primary surgical treatment with curative intent: T- and N-classification, resection margin status, lymphangiosis, vascular invasion, grading, extracapsular extension (ECE), HPV status, sex, primary site and smoking history. Results are presented in Table 4.

**Table 3:** Cox regression analysis of known prognostic parameters and MAGE expression in the whole cohort

Factor		*n*	HR	95% CI		*p* value
T	T1	163	1	1	1	
	T2	168	2.216	1.485	3.305	< 0.001
	T3	107	2.999	1.985	4.532	< 0.001
	T4	79	4.766	3.134	7.246	< 0.001
	T1	163	1	1	1	
	T2-4	354	2.929	2.055	4.174	< 0.001
	T1-2	331	1	1	1	
	T3-4	185	2.368	1.815	3.089	< 0.001
	T1-3	438	1	1	1	
	T4	79	2.540	1.867	3.457	< 0.001
N	N0	256	1	1	1	
	N1	76	1.905	1.282	2.832	0.001
	N2a	25	1.820	0.99	3.346	0.054
	N2b	99	2.528	1.782	3.587	< 0.001
	N2c	46	2.629	1.705	4.054	< 0.001
	N3	10	2.651	1.155	6.087	0.021
	N0	254	1	1	1	
	N1	78	1.723	1.156	2.568	0.008
	N2	170	2.374	1.756	3.208	< 0.001
	N3	10	2.587	1.127	5.937	0.025
	N0	254	1	1	1	
	N+	258	2.168	1.639	2.868	< 0.001
M	M0	500	1	1	1	
	M1	19	2.62	1.523	4.505	< 0.001
Grading	1	18	1	1	1	
	2	274	1.223	0.537	2.786	0.631
	3	126	1.628	0.704	3.763	0.254
Treatment	Surgery	446	1	1	1	
	Non-surgical	66	3.012	2.189	4.146	< 0.001
HPV	HPV DNA −	481	1	1	1	
	HPV DNA +	38	0.382	0.189	0.775	0.008
	HPV, p16 −	488	1	1	1	
	HPV + p16 +	26	0.414	0.184	0.932	0.033
Sex	female	130	1	1	1	
	male	389	0.793	0.589	1.068	0.127
Primary Site	Larynx	169	1	1	1	
	Oral Cavity	120	2.324	1.602	3.37	< 0.001
	Oropharynx	167	1.787	1.255	2.545	0.001
	Hypopharynx	62	1.684	1.056	2.686	0.029
Smoking	Non-Smoker	47	1	1	1	
	Smoker	368	1.345	0.777	2.326	0.290
	Non-smoker	47	1	1	1	
	< = 10 py	25	1.044	0.452	2.412	0,920
	> 10 < 20	44	0.931	0.437	1.98	0,852
	> 20 < 30	75	1.141	0.596	2.186	0,690
	> 30 < 40	81	1.134	0.599	2.147	0,700
	> 40	143	1.809	1.020	3.208	0,043
	Non-smoker	47	1	1	1	
	< median (36)	162	1.025	0.569	1.847	0,935
	> median (36)	206	1.625	0.927	2.848	0,090
	Non-Smoker	47	1	1	1	
	< 40 py	163	1.017	0.564	1.834	0,954
	> = 40 py	205	1.636	0.933	2.867	0,086
pan-MAGE	negative	237	1	1	1	
	positive	162	1.234	0.913	1.669	0.172
	negative	237	1	1	1	
	cyt OR nuc	83	1.119	0.760	1.647	0,570
	cyt + nuc	79	1.356	0.938	1.961	0,105
	MEI < 0.1	269	1	1	1	
	MEI 0.1–0.39	86	1.107	0.768	1.597	0,585
	MEI 0.4–0.69	18	1.582	0.827	3.025	0,165
	MEI 0.7–1	9	1.301	0.53	3.189	0,566
MAGE-A3/A4	negative	244	1	1	1	
	positive	155	1.320	0.976	1.786	0.072
	negative	245	1	1	1	0.129
	cyt OR nuc	73	1.242	0.835	1.846	0.285
	cyt + nuc	81	1.432	0.998	2.054	0.051
	MEI < 0.1	281	1	1	1	
	MEI 0.1–0.39	70	1.156	0.776	1.720	0.476
	MEI 0.4–0.69	22	1.147	0.618	2.131	0.663
	MEI 0.7–1	5	2.135	0.787	5.793	0.136
MAGE-A1	negative	324	1	1	1	
	positive	76	1.011	0.691	1.478	0.957

The comparators for each variable are signified by a HR of 1.

Abbreviations: T = T-classification, N = N-classification, M = M-classification, ECE = extracapsular extension, L = Lymphangiosis, V = vascular tumor emboli, R = resection margin status, HPV = human papilloma virus. *n* = absolute number of cases with available data, HR = hazard ratio, CI = confidence interval.

**Table 4:** Cox regression analysis of known prognostic markers and MAGE expression in the surgical cohort

Factor		*n*	HR	95% CI		*p* value
T	T1	157	1	1	1	
	T2	155	2.401	1.582	3.643	< 0.001
	T3	83	2.952	1.877	4.644	< 0.001
	T4	41	2.965	1.734	5.070	< 0.001
	T1	157	1	1	1	
	T2-4	279	2.651	1.815	3.873	< 0.001
	T1-2	312	1	1	1	
	T3-4	124	1.829	1.335	2.507	< 0.001
	T1-3	395	1	1	1	
	T4	41	1.594	1.016	2.500	0.042
N	N0	236	1	1	1	
	N1	66	1.856	1.199	2.873	0.006
	N2a	16	1.103	0.444	2.738	0.833
	N2b	79	2.633	1.774	3.908	< 0.001
	N2c	36	2.622	1.604	4.288	< 0.001
	N3	3	1.325	0.184	9.550	0.780
	N0	233	1	1	1	
	N1	69	1.730	1.118	2.679	0.014
	N2	131	2.344	1.664	3.302	< 0.001
	N3	3	1.308	0.181	9.429	0.790
	N0	233	1	1	1	
	N+	203	2.100	1.534	2.876	< 0.001
R	R0	326	1	1	1	
	R1	30	1.466	0.828	2.597	0.190
	R2	4	2.056	0.507	8.333	0.313
	R0	326	1	1	1	
	R+	34	1.524	0.892	2.606	0.123
L	L0	137	1	1	1	
	L1	60	2.368	1.477	3.798	< 0.001
V	V0	173	1	1	1	
	V1	14	1.848	0.843	4.052	0.125
Grading	1	18	1	1	1	
	2	238	1.106	0.483	2.533	0.811
	3	112	1.409	0.604	3.285	0.427
ECE	–	109	1	1	1	
	+	61	1.697	1.094	2.631	0.018
HPV	HPV DNA −	403	1	1	1	
	HPV DNA +	34	0.373	0.165	0.844	0.018
	HPV DNA–OR p16 −	409	1	1	1	
	HPV + AND p16 +	26	0.485	0.215	1.097	0.082
Sex	female	111	1	1	1	
	male	326	0.798	0.568	1.123	0.196
Primary Site	Larynx	142	1	1	1	
	Oral Cavity	110	3.213	2.09	4.940	< 0.001
	Oropharynx	134	2.044	1.327	3.148	0.001
	Hypopharynx	51	1.748	0.984	3.105	0.057
Smoking	Non-Smoker	42	1	1	1	
	Smoker	300	1.109	0.61	2.018	0.734
	Non-smoker	42	1	1	1	
	< = 10 py	21	0.84	0.315	2.238	0.727
	> 10 < 20	40	0.781	0,337	1.808	0.564
	> 20 < 30	61	0.857	0.406	1.813	0.687
	> 30 < 40	62	0.88	0.423	1.827	0.731
	> 40	116	1.587	0.847	2.976	0.150
	Non-smoker	42	1	1	1	
	< median (36)	136	0.825	0.429	1.586	0.564
	> median (36)	164	1.368	0.739	2.533	0.318
	Non-Smoker	42	1	1	1	
	< 40 py	137	0.818	0.425	1.572	0.547
	> = 40 py	163	1.379	0.745	2.553	0.306
pan-MAGE	negative	202	1	1	1	
	positive	143	1.454	1.037	2.040	0.030
	negative	202	1	1	1	
	cyt OR nuc	73	1.354	0.883	2.075	0.165
	cyt + nuc	70	1.556	1.034	2.343	0.034
	MEI < 0.1	238	1	1	1	
	MEI 0.1–0.39	72	1.001	0.654	1.533	0.995
	MEI 0.4–0.69	16	1.896	0.953	3.772	0.068
	MEI 0.7–1	9	1.513	0.614	3,729	0.368
MAGE-A3/A4	negative	207	1	1	1	
	positive	139	1.511	1.077	2.119	0.017
	negative	208	1	1	1	
	cyt OR nuc	67	1.436	0.931	2.216	0.102
	cyt + nuc	71	1.636	1.094	2.448	0.017
	MEI < 0.1	243	1	1	1	
	MEI 0.1–0.39	62	1.293	0.834	2.004	0.251
	MEI 0.4–0.69	21	1.244	0.646	2.393	0.514
	MEI 0.7–1	5	2.622	0.96	7.149	0.060
MAGE-A1	negative	279	1	1	1	
	positive	67	0.853	0.565	1.288	0.450

The comparators for each variable are signified by a HR of 1.

Abbreviations: T = T-classification, N = N-classification, ECE = extracapsular extension, L = Lymphangiosis, V = vascular tumor emboli, R = resection margin status, HPV = human papilloma virus. *n* = absolute number of cases with available data, HR = hazard ratio, CI = confidence interval.

### Correlation of MAGE protein expression with known prognostic markers

A Pearson correlation revealed significant correlations between pan-MAGE expression and T-classification, N-status, HPV-status and smoking, but all correlation coefficients were *R* < 0.2. For MAGE-A3/A4 a significant correlation was found with T-classification, HPV-status and smoking. All correlation coefficients were *R* < 0.2. MAGE-A1 was significantly correlated with T-classification, N-status and HPV-status. All correlation coefficients were *R* < 0.2. All correlation coefficients and significance values are provided in Table 5.

**Table 5:** Pearson correlation of MAGE expression with known prognostic markers

		T	N	M	ECE	L	V	Grading	R	HPV-DNA	HPV-DNA, p16	Sex	Primary Site	Smoking
pan-MAGE (M3H67)	Correlation Coefficient	0.148	0.112	(-)0.064	0.047	0.102	0.069	0.062	0.02	(-)0.115	(-)0.172	(-)0.02	0.07	0.158
	p-value	0.002**	0.023*	0.188	0.547	0.207	0.408	0.251	0.73	0.018*	<0.001***	0.674	0.152	0.004**
	number of cases	420	415	423	167	155	147	346	311	424	420	424	424	326
MAGE-A3/A4 (57B)	Correlation Coefficient	0.107	0.074	(-)0.032	(-)0.004	0.1	0.106	0.065	0.049	(-)0.14	(-)0.146	(-)0.012	0.082	0.113
	p-value	0.029*	0.131	0.506	0.964	0.216	0,2	0.229	0.385	0.004**	0.003**	0.799	0.092	0.042*
	number of cases	420	415	423	167	155	147	346	312	424	420	424	424	326
MAGE-A1 (MA454)	Correlation Coefficient	0.168	0.148	0.034	0.027	0.079	0.078	(-)0.001	0.08	(-)0.079	(-)0.096	(-)0.06	0.089	0.035
	p-value	0.001**	0.002**	0.488	0.731	0.33	0.349	0.984	0.157	0.105	0.049*	0.216	0.065	0.528
	number of cases	421	416	424	167	155	147	346	312	425	421	425	425	327
														

Numbers of cases represent those for which data for both compared variables were available.

Abbreviations: T=T-classification, N=N-classification, M=M-classification, ECE=extracapsular extension, L=Lymphangiosis, V=vascular tumor emboli, R=resection margin status, HPV=human papilloma virus.

Due to the very low correlation coefficients, the significant correlations with known prognostic markers can be considered irrelevant.

### Prognostic value of MAGE protein expression

**Figure 4:**
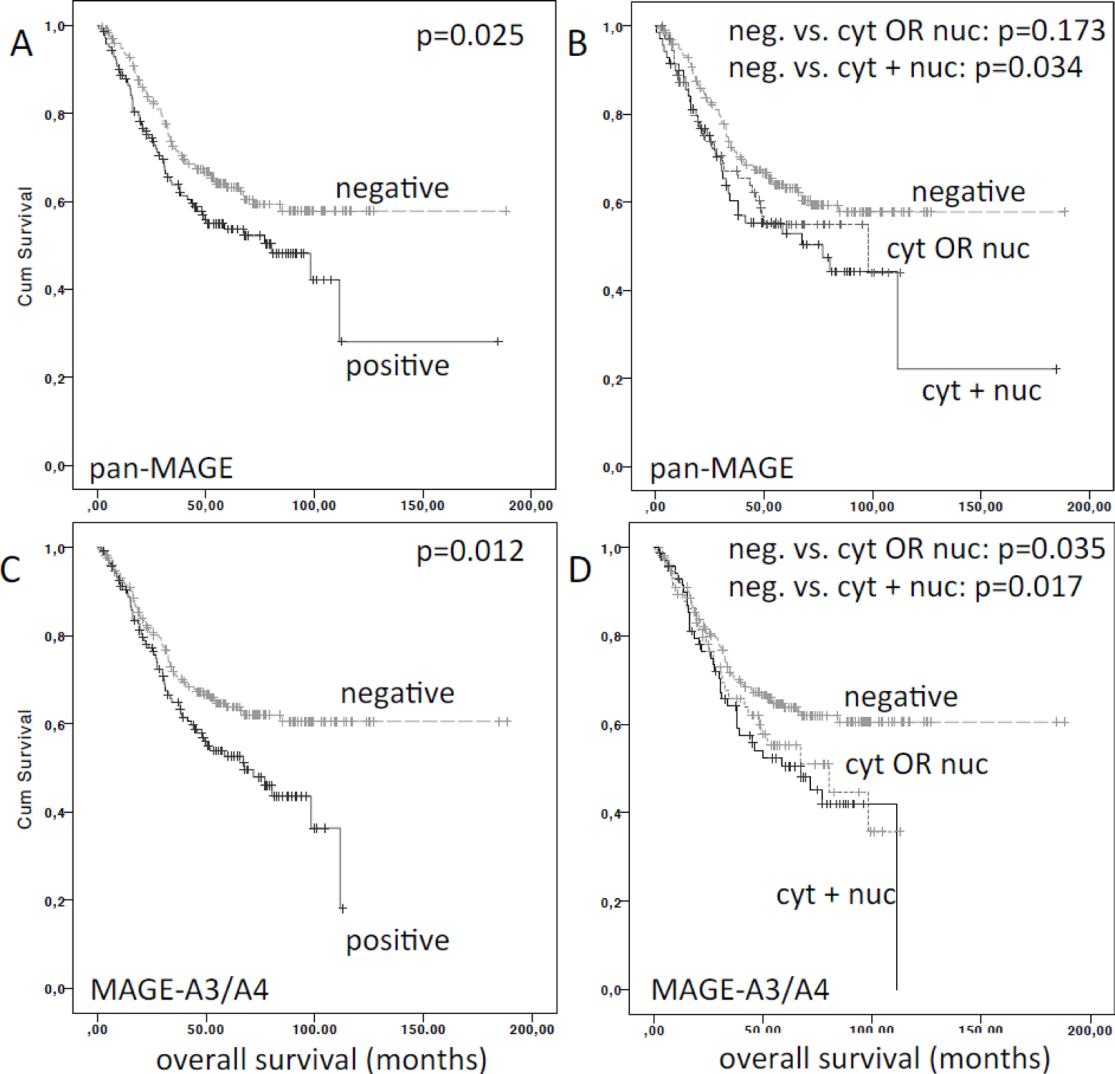
Overall survival (OS) of patients stratified by MAGE expression Overall survival was significantly reduced for patients expressing (**A**) pan-MAGE (M3H67) and (**C**) MAGE-A3/A4 (57B). In panel (**B**) and (**D**) the respective patients were stratified by the expression pattern of (B) pan-MAGE and (D) MAGE-A3/A4. The *p*-values for pairwise comparisons are indicated.

In order to validate our previously reported prognostic impact of pan-MAGE and MAGE-A3/A4 expression on OS in surgically treated patients [10], we performed survival analyses for OS using the Kaplan-Meier method. Patients with available survival information were filtered for patients who received primary surgical treatment with curative intent. OS was significantly lower for patients showing expression of pan-MAGE (mean OS pan-MAGE_neg_ (*n* = 202) = 122.8 months vs. mean OS pan-MAGE_pos_ (*n* = 143) = 88.7 months, *p* = 0.025) and MAGE-A3/A4 (mean OS MAGE-A3/A4_neg_ (*n* = 208) = 125.5 months vs. mean OS MAGE-A3/A4_pos_ (*n* = 138) = 66.9 months, *p* = 0.012) (Figure 4A, 4C).

Next, we evaluated the impact of the respective expression pattern on OS. Compared to negative cases, OS for pan-MAGE_cytORnuc_ (*n* = 73, mean OS=71.3 months) was lower, but did not reach significance (*p* = 0.173). In contrast, OS in pan-MAGE_cyt+nuc_ (*n* = 70, mean OS=82.9 months) was significantly reduced (*p* = 0.034). Compared to negative cases, OS for MAGE-A3/A4_cytORnuc_ (*n* = 67, mean OS = 68.6 months) was lower, but did not reach significance (*p* = 0.111). In contrast, OS in MAGE-A3/A4_cyt+nuc_ (*n* = 71, mean OS=65.9 months) was significantly lower (*p* = 0.017) (Figure 4B, 4D).

In the subgroup of oral cavity primaries, mean OS was significantly lower for pan-MAGE_cyt+nuc_ (31.5 months, *p* = 0.004) as well as pan-MAGE_cytORnuc_ (29.7 months, *p* = 0.049) compared to negative cases (72.7 months). Mean OS was also significantly lower for MAGE-A3/A4_cyt+nuc_ (28.6 months, *p* = 0.009) compared to negative cases (70.8 months). Mean OS of MAGE-A3/A4_cytORnuc_ was also lower (48.2 months), but did not reach significance compared to negative cases (*p* = 0.246).

In the subgroup of oropharyngeal primaries, mean OS was not significantly different for pan-MAGE_cyt+nuc_ (93.9 months, *p* = 0.125) or pan-MAGE_cytORnuc_ (64.2 months, *p* = 0.281) compared to negative cases (87.0 months). However, mean OS was lower for MAGE-A3/A4_cyt+nuc_ (55.7 months, *p* = 0.08) compared to negative cases (125.9 months). Mean OS of MAGE-A3/A4_cytORnuc_ was significantly lower (58.7 months, *p* = 0.043) compared to negative cases.

In the subgroups of hypopharyngeal and laryngeal primaries, the survival was not significantly lower for pan-MAGE or MAGE-A3/A4 expression (*p* > 0.05).

None of the evaluated antigens was significantly associated with disease-free survival (DFS; data not shown).

A multivariate Cox regression analysis for OS taking into account T-classification, HPV-status (DNA), primary tumor site, pan-MAGE expression and MAGE-A3/A4 expression was performed. For this analysis 344 patients were available. Multivariate cox regression revealed the following markers as independently prognostic: T-classification (T2 HR = 2.2; T3 HR = 3.02; T4 HR = 3.3), HPV-status (HPV negative: HR = 3.5), primary site compared to laryngeal primaries (oral cavity: HR = 3.2; oropharynx: HR = 1.7; hypopharynx: HR = 1.1) and MAGE-A3/A4 expression (HR = 1.4).

### TCGA control cohort

As control cohort, TCGA data were analyzed for OS. For the gene products recognized by the M3H67 antibody (pan-MAGE: MAGE-A1, -A3, -A4, -A8, -A10, -B2, -C2), 117/522 (22 %) of cases had upregulated mRNA expression for any of the genes. OS was significantly reduced for cases with increased expression of pan-MAGE gene mRNA (negative cases: 64.78 months vs. positive cases: 30.91 months; *p* = 0.0057). For the gene products recognized by the 57B antibody (MAGE-A3/A4: MAGE-A3, -A4, -A6, -A12), 93/522 (18 %) of the cases showed upregulated mRNA expression for any of the genes. OS was also significantly reduced for cases expressing MAGE-A3/A4 genes (negative cases: 57.88 months vs. positive cases: 30.91 months; *p* = 0.0094). Survival curves for the queried genes from the TCGA dataset are given in Supplementary Figure 1. DFS was not significantly different for any of the queried gene sets in the TCGA cohort (pan-MAGE: *p* = 0.679; MAGE-A3/A4: *p* = 0.361; data not shown).

## DISCUSSION

This is to the best of our knowledge, the first study examining the expression of MAGE antigens in a large cohort of HNSCC patients with focus on the comparison of expression frequency and expression intensity in paired samples of primary tumors, lymph node metastases, and recurrences.

The MAGE expression frequency of pan-MAGE and MAGE-A1 in PT in this analysis was comparable to our previously published results in a similarly large independent cohort of HNSCC patients from Hamburg, Germany, but MAGE-A3/A4 expression was found more frequently (TMA Hamburg: 27.7% vs. TMA Bonn: 39%) [10].

In this analysis, a significant correlation with known prognostic markers was found. However, the correlation coefficients were weak (*R* < 0.2). Therefore we consider this correlation irrelevant. Previously, two other studies have reported no significant correlation with clinicopathological criteria [10, 13]. In contrast to our results, one group reported a higher prevalence of MAGE-A3/A4 expression (57B) in T3/T4 laryngeal cancer and of MAGE-A1 (MA454) and pan-MAGE (M3H67) in cases which developed regional recurrence, but the corresponding correlation coefficients were not reported [11].

The main goal of this study was the comparison of MAGE expression in PT, LNM and RD. The use of digital pathology enabled us to obtain continuous values for the expression intensity from 0 to 1. This way we were able to obtain a mean expression intensity (MEI) for the available triplicates of each tumor sample and to compare the median of the MEI between the three groups. Significant differences between the MEI of PT, LNM and RD were observed for pan-MAGE and MAGE-A1. The graphed data (Figure 2) indicate an increase of the MEI when comparing PT with LNM or RD, respectively. This may indicate an increasing expression intensity of MAGE during tumor evolution although the expression frequencies were similar between PT, LNM and RD (Table 2). Mechanistic data in HNSCC cell lines implied reduced responses to different chemotherapeutic drugs [16]. MAGE-A has been shown to impair p53 function [18] which may lead to reduced chemosensitivity as well as reduced radiosensitivity [19]. Another group showed increased MAGE-A3 RNA expression in a cancer-stem cell population in bladder cancer [15]. Zamuner et al. reported reduced incidence of recurrence in MAGE-A3/A6 positive tumors [20]. However, Zamuner et al. did not assess MAGE expression in samples of recurrent tumors. Thus, these data do not contradict our results that in recurrent tumors a higher median MEI of pan-MAGE and MAGE-A1 was found.

The comparison of the MEI in paired samples from primary tumors and recurrences revealed a significantly different MEI distribution. A higher number of recurrences with a MEI ≥ 0.1 and a MEI < 0.1 in the respective PT was observed. Published data from gastrointestinal stromal tumors identified CTA expression as a marker for early recurrence [21], which was validated prospectively in a subsequent study [22]. These facts further support our hypothesis that MAGE expression intensity increases in recurrent cancer. Most published data indicate a selection advantage of MAGE expression for cancer cells and support our hypothesis of differential MAGE expression during tumor evolution. In clinical trials with MAGE-specific vaccination for RD, the expression should therefore be assessed in a sample from the recurrent tumor and not from archived tissue.

The analysis of paired samples of PT and LNM enables us to draw further conclusions. Although the MEI in paired samples of PT and LNM was well concordant, mismatches were also observed in ≈20% for pan-MAGE and MAGE-A3/A4.

Regarding our previously reported prognostic implications of pan-MAGE and MAGE-A3/A4, we were able to validate the lower OS of pan-MAGE and MAGE-A3/A4 expressing patients as well as the impaired survival in cases with cyt+nuc expression in this independent cohort [10]. However, concerning the prognostic value of pan-MAGE and MAGE-A3/A4, the impact on prognosis in this cohort was smaller than in our previously analyzed cohort. This may be a result of the different distribution of primary sites and patient characteristics in the two cohorts. Compared to the TMA Hamburg, this cohort consisted of a smaller proportion of oral cavity cancers and a larger number of oropharyngeal tumors [10]. One of the main prognostic factors in HNSCC is HPV. However, in the TMA Bonn, the rate of HPV DNA positive cases was low with 39/552 (7%) cases. There was a lower frequency of MAGE expression in HPV positive cases compared to negative cases. But in this cohort, the overall number of HPV DNA positive cases (*n* = 39) is too low to generalize these findings regarding MAGE expression in HPV positive HNSCC. The prognostic impact of MAGE expression on OS was pronounced in the oral cavity cohort in both independent patient cohorts and oral cavity squamous cell carcinoma is rarely associated with HPV infection (TMA Bonn: 4.7%). Due to the low rate of HPV positive cases and results of the multivariate Cox regression analysis, we can rule out that the prognostic disadvantage of pan-MAGE and MAGE-A3/A4 expression was actually based on the survival advantage of HPV positive patients. On the contrary, a subgroup analysis of HPV positive patients revealed that there was a non-significant trend to improved survival of MAGE-positive cases in the HPV positive subgroup (no events in the MAGE-positive patients compared to a death rate of 20% in MAGE negative cases after 5 years, Supplementary Figure 2). This may be due to increased immune-surveillance in HPV positive HNSCC [23–25].

Furthermore the laryngeal cohort from Bonn had a higher rate of early glottic laryngeal primaries known to have a good prognosis and few events to analyze. Thus, in the laryngeal subgroup of patients, we did not find reduced survival for neither pan-MAGE nor MAGE-A3/A4 expression. These facts may explain why we were not able to confirm pan-MAGE expression as an independent prognostic marker by multivariate cox regression. However, MAGE-A3/A4 expression was confirmed as an independent prognostic marker in this cohort.

The analysis of the independent cohort from the TCGA databank further confirmed the prognostic significance of pan-MAGE and MAGE-A3/A4 genes using RNA sequencing data. Thus, the prognostic implications are based on >1000 individual patients and two different methods (RNA sequencing, IHC).

Another group reported better DFS for MAGE-A3/A6 positive compared to negative patients based on polymerase chain reaction data for MAGE-A3/A6 RNA [20]. MAGE-A6 is a homologue protein to MAGE-A3 and the 57B antibody binds to MAGE-A6 as well, as we have previously shown by ELISA [10]. In our data no significant differences for DFS according to MAGE expression were evident. However, there was a tendency to reduced DFS for MAGE-A3/A4 positive cases, which did not reach significance. The TCGA data also support our finding that DFS is not significantly different based on the expression of MAGE. Thus, the impact of MAGE expression on the incidence of recurrence and DFS will have to be finally validated prospectively. One reason for the discrepancies between these different cohorts may lie in the extent of CTA specific immunity and whether/how these immune reactions were counterbalanced by immune modulatory checkpoints. However, a prospective validation of the prognostic impact is needed to establish MAGE expression as a prognostic marker for clinical routine.

Antigens of the MAGE family are interesting shared targets for vaccination due to their tumor specificity and immunogenicity [8]. For HNSCC, no large trials with a relevant patient number have been performed and published to date. However, in a large phase III MAGE-A3 vaccination trial for NSCLC, no improvement of DFS was found [26]. Central tolerance to these self antigens is discussed as an explanation for the observation of low-avidity T cells to CTA [27, 28] .

At the same time, new opportunities for immunotherapy have emerged with the successful development of different immune checkpoint modulators targeting inhibitory molecules such as cytotoxic T lymphocyte-associated antigen 4 (CTLA-4), programmed death 1 (PD-1) and its ligand PD-L1 or immune stimulatory molecules such as OX40 or CD137. Data from melanoma treated with immune checkpoint modulators suggest that a high mutational load is associated with a good clinical response to immune checkpoint blockade [29]. This is explained by a higher probability of mutations leading to the expression of so-called mutational neoantigens which can be recognized by the immune system [29–31]. In the studies demonstrating immune responses to neoantigens in patients treated with immune checkpoint blockade, other non-mutational antigens such as cancer testis antigens (CTA) were, due to the methodology of the antigen screening process, neglected thus far. However, in melanoma patients treated with ipilimumab an increased rate of NY-ESO-1 specific immunity has been associated with improved clinical benefit of treatment, especially in patients developing both NY-ESO-1 specific antibodies and specific CD8 T cells [32]. Thus, the addition of an antigen-specific treatment in the form of a vaccine may increase the rate of responders to immune checkpoint targeted therapy. The combination of immune-checkpoint modulators and a vaccine, composed of several different CTA, may be an opportunity to improve the benefit of CTA vaccination. To evaluate whether this strategy may be a valid approach to improve OS of CTA-positive HNSCC patients, a systematic evaluation of spontaneous immunity to CTA in HNSCC patients before and during conventional curative treatment should be performed.

Due to tumor heterogeneity, the tissue sampling process for TMA generation may represent a bias. We reduced sampling bias by sampling three separate cores of each tumor sample in the TMA construction progress. Furthermore, the addition of digital pathology to microscopic evaluation enabled us to address tumor heterogeneity by obtaining MEI as a continuous semiquantitative variable for protein expression derived from the analysis of the three triplicates.

This analysis was retrospectively performed. Thus, we cannot exclude a selection bias. Selection bias was addressed by the selection of every patient with available tissue specimen who was treated for HNSCC between 1997 and 2011 at the University Hospital of Bonn.

In conclusion, the prognostic disadvantage of MAGE expression and the increasing MEI in recurrent disease imply that the intrinsic functions of MAGE genes support an aggressive malignant phenotype. It seems that during conventional therapy, the immune system fails to outbalance this aggressiveness in MAGE positive tumors, resulting in reduced OS. A systematic analysis of MAGE-specific immunity and MAGE gene function in HNSCC is needed to shed light on the causal relationship of the prognostic disadvantage. Also, the impact of MAGE expression in relation to HPV status needs to be validated in a larger cohort of HPV positive patients. Immunotherapy, incorporating immune checkpoint modulation in combination with a vaccine may improve MAGE-specific immunity and improve OS. Still, prospective trials are needed to determine the value of MAGE expression as a prognostic marker for clinical routine.

## MATERIALS AND METHODS

### Patients/tissue microarray

Patients were treated according to local treatment guidelines between 1997 and 2011 at the University Hospital of Bonn. A tissue microarray (TMA) was constructed from archived formalin-fixed paraffin-embedded tissue samples after clinical diagnostics were finished. Representative 0.6mm cores were assembled into TMA blocks. The TMA included 552 primary tumors, 219 lymph node metastases and 75 recurrences. Each tumor specimen was sampled in triplicates. The study was approved by the internal review board of the University Hospital of Bonn (#174/13).

For an independent control cohort, TCGA database was accessed on May 1^st^, 2016. At this time 522 HNSCC cases with RNA Seq V2 data were available for analysis. The cBioPortal for Cancer Genomics (cbioportal.org) was used to access and analyze TCGA database [33, 34]. Antigens for the query were based on the previously established antibody specificities of the respective antibodies. M3H67 (pan-MAGE): MAGE-A1, -A3, -A4, -A8, -A10, -B2, -C2 and 57B (MAGE-A3/A4): MAGE-A3, -A4, -A6, -A12. The z-score was set at 2.0.

### Immunohistochemistry

Immunohistochemistry (IHC) for MAGE-antigens was performed using three antibodies (pan-MAGE: clone M3H67, MAGE-A3/A4: clone 57B, MAGE-A1: clone MA454) on 4μm TMA sections as described previously [10, 35]. Antibody specificities have also been previously analyzed by enzyme linked immunosorbent assay [10]. Based on these previously reported results, we will refer to M3H67 reactivity as pan-MAGE expression, to 57B as MAGE-A3/A4 and to MA454 as MAGE-A1. The following primary antibody concentrations were used: M3H67 = dilution of 1:3324 (0.74 μg/ml), 57B = dilution of 1:150, MA454 = dilution of 1:589 (5.7 μg/ml).

Each tumor core was microscopically evaluated by two independent investigators and was scored as positive for the respective antigen if specific staining was present in ≥ 10% of tumor cells. The tumor sample was classified positive if at least one core of the triplicate was scored as positive in agreement between the two investigators. Additionally, the expression pattern was noted (cytoplasmic OR nuclear (cytORnuc), cytoplasmic AND nuclear (cyt+nuc)).

TMAs were then digitized using the Zeiss MIRAX DESK scanner. Staining intensity per tumor area was determined using a semiautomated, quantitative image analysis software (Definiens Architect XD 1.2, Definiens, Munich, Germany) in order to obtain a continuous spectrum of average staining intensity per tumor area in arbitrary units (maximum range of readout 0.0–1.0) for the complete tumor areas of each core. Thus, expression intensity per area contains both, the stained area of the tumor in relation to the total tumor area and the expression intensity averaged over the total tumor area. The mean expression intensity (MEI) was derived from the mean of up to three available cores for each tumor specimen. This way intratumoral heterogeneity was addressed by taking into account both, the stained tumor area and the staining intensity in relation to the total tumor area for each tumor in three separate tumor cores. The MEI was determined separately for the triplicates of primary tumors, lymph node metastases and recurrences.

### Statistics

*IBM SPSS statistics version 21.0* was used for statistical analysis and graphing of results unless otherwise indicated. Cox regression analysis for OS was performed to determine hazard ratios (HR) with a 95% confidence interval (CI). For multivariate cox regression backward likelihood ratio was applied. A Pearson correlation was used to quantify correlations between MAGE expression and known prognostic markers. OS was defined as the time interval from diagnosis until death. OS was analyzed using the Kaplan-Meier method. Groups were compared by log-rank-test. TCGA data were analyzed for OS using the analysis tool cbioportal.org [33, 34].

MEI of primary tumors, lymph node metastases and recurrences was compared and graphed using Kruskal-Wallis test with *Graph Pad Prism version 6*. MEI in paired samples was compared using the Wilcoxon signed rank test for related samples. Significance was assumed for *p* < 0.05.

## SUPPLEMENTARY MATERIALS FIGURES AND TABLES



## Abbreviations

CI: confidence interval
NSCLC: non-small cell lung cancer
CTA: cancer-testis antigens
nuc: nuclear
cyt: cytoplasmic
OS: overall survival
ECE: extracapsular extension
PT: primary tumor
HNSCC: head and neck squamous cell carcinoma
RD: recurrent disease
HPV: human papillomavirus
RFS: recurrence-free survival
HR: hazard ratio
RNA: ribonucleic acid
LNM: lymph node metastasis
TCGA: The Cancer Genome Atlas
MAGE: melanoma associated antigen
TMA: tissue micro-array
MEI: mean expression intensity
