# Supplementary material for: MAGE expression in head and neck squamous cell carcinoma primary tumors, lymph node metastases and respective recurrences-implications for immunotherapy
Source: Oncotarget. 2017 Jan 27;8(9):14719–35. doi: 10.18632/oncotarget.14830 (PMC5362438; doi:10.18632/oncotarget.14830)
Supplement: Supplementary file 1 [file oncotarget-08-14719-s001.pdf]

MAGE expression in head and neck squamous cell carcinoma primary tumors, lymph node metastases and respective recurrences-implications for immunotherapy

Supplementary Materials

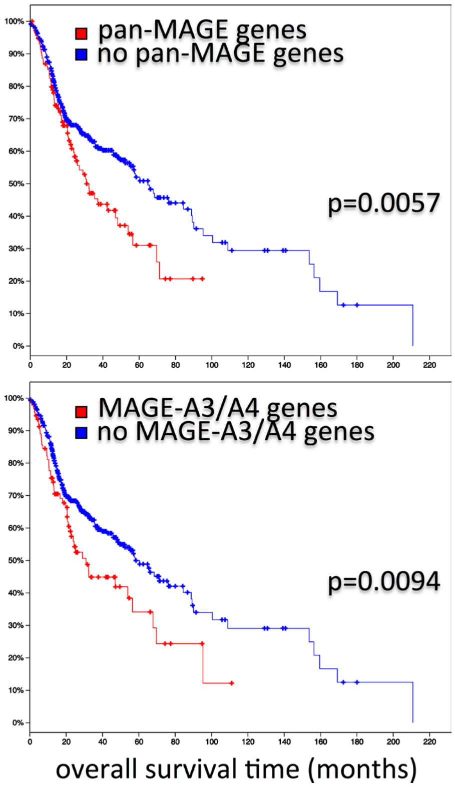

Supplementary Figure 1: Overall survival based on MAGE gene signatures in the TCGA cohort. TCGA data as analyzed for OS using the analysis tool cbioportal.org. The upper Kaplan-Meier curve shows OS for tumors expressing upregulated mRNA of the genes recognized by the M3H67 antibody (pan-MAGE genes: MAGE-A1, -A3, -A4, -A8, -A10, -B2, -C2). Overall, 517 cases with data were available for this analysis. Sixty of 116 cases with upregulated pan-MAGE genes and 160/401 cases without upregulated pan-MAGE genes had events to analyze. The lower Kaplan-Meier curve shows OS for tumors expressing upregulated mRNA of the genes recognized by the 57B antibody (MAGE-A3/A4: MAGE-A3, -A4, -A6, -A12). Overall, 517 cases with data were available for this analysis. Forty-eight of 92 cases with upregulated MAGE-A3/A4 genes and 172/425 cases without upregulated MAGE-A3/A4 genes had events to analyze. The results shown here are in whole based upon data generated by TCGA Research Network: http://cancergenome.nih.gov/.

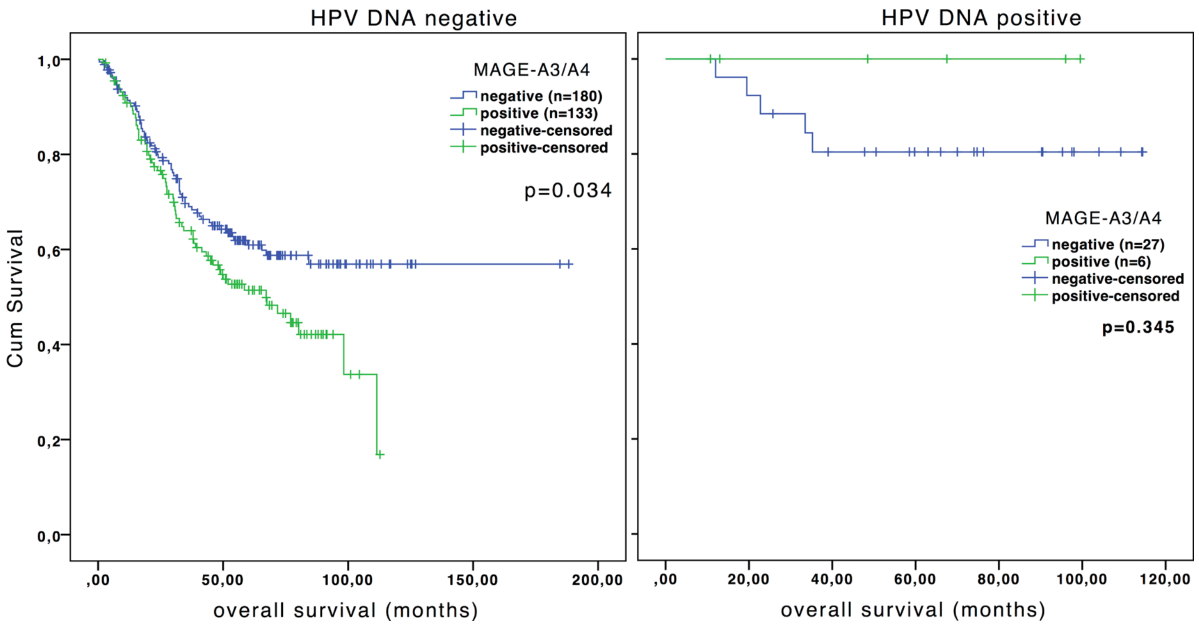

Supplementary Figure 2: Subgroup analysis of the prognostic impact of MAGE-A3/A4 expression in the HPV negative subgroup and the HPV positive subgroup based on HPV-DNA status. MAGE-A3/A4 expression was significantly associated with lower survival in the HPV negative subgroup (p = 0.034), but not in the HPV positive subgroup (p = 0.345). Notably, in the HPV positive subgroup, no events were registered in the MAGE-A3/A4 positive patients, whereas 20% of MAGE-A3/A4 negative patients had an event.
